# Municipal water fluoridation, adolescent IQ, and cognition across the life course: Evidence from the Wisconsin Longitudinal Study

**DOI:** 10.1073/pnas.2536005123

**Published:** 2026-04-13

**Authors:** John Robert Warren, Gina Rumore, Kamil Sicinski, Pamela Herd, Michal Engelman

**Affiliations:** ^a^Institute for Social Research and Data Innovation, University of Minnesota, Minneapolis, MN 55455; ^b^Center for Demography of Health and Aging, University of Wisconsin, Madison, WI 53706; ^c^Gerald Ford School of Public Policy, University of Michigan, Ann Arbor, MI 48109

**Keywords:** fluoride, cognition, cohort

## Abstract

We investigate associations between community water fluoridation (CWF), adolescent IQ, and cognition across the life course using representative data from the US state of Wisconsin. Exposure is inferred from historical records on community water fluoridation; adolescent IQ is ascertained from state testing records; and cognition in later life is assessed as part of the Wisconsin Longitudinal Study [P. Herd *et al.*, *Int. J. Epidemiol.* **43**, 34–41 (2014).]. In contrast to studies cited in recent decisions to end CWF in Utah, Florida, and elsewhere, we find no evidence that CWF is negatively associated with adolescent IQ or adult cognitive functioning.

A recent analysis (1) finding a negative relationship between fluoride exposure and adolescent IQ was prominently cited in decisions to end community water fluoridation (CWF) in parts of the United States. However, the quality and salience of that evidence have been questioned (2, 3). Most notably, the bulk of the evidence presented by Taylor et al. (1) concerned extremely high dosages of fluoride—far exceeding levels relevant to CWF policy discussions. None of their evidence came from population-representative samples; most failed to account for selection into treatment. None of the research was conducted using data collected in the United States.

Evidence about the association between adolescent IQ and fluoride exposures at levels recommended for CWF has been mixed (4); until recently, there was no evidence about fluoride exposure and later-life cognition. Warren et al. (2) recently provided the first analysis of the relationship between CWF and cognition across the life course, the first analysis using population-representative data from the United States, and the first analysis carefully accounting for confounding of the association between fluoride exposure and adolescent cognition. They found no evidence for negative relationships between CWF and adolescent or adult cognition.

We replicate and extend Warren et al.’s (2) analyses, improving upon them in two important ways. First, whereas they estimated the impact of fluoride exposure on adolescent academic achievement, we model effects on adolescent IQ. Second, whereas they were forced to assume that secondary school students lived their entire lives in their high school’s community, we observe geographic mobility across childhood (and thus treatment consistency).

## Results

Table 1 describes a) outcome measures of IQ at age 16 and of cognition at several ages in later life and b) individual- and school-level measures that may confound associations between CWF and cognition. While cognitive outcomes are highest for Wisconsin Longitudinal Study (WLS) (5) participants who were first exposed to CWF in late adolescence, it is also true that socioeconomic advantage is highest among those sample members.

**Table 1.** Descriptive statistics, by exposure category

Measure (WLS Variable Name)	No Exposure (n = 3,614)		Exposed from Birth (n = 2,595)		Exposed from Age 8 (n = 2,087)		Exposed from Age 14 (n = 2,021)	
	Mean	(SD)	Mean	(SD)	Mean	(SD)	Mean	(SD)
Cognitive Outcomes								
Age 16—IQ (gwiiq_bm)	−0.07	(1.00)	0.00	(1.02)	0.01	(1.01)	0.11	(0.96)
Age 53—WAIS Similarities (ri001re)	−0.02	(1.01)	−0.04	(1.00)	−0.02	(0.99)	0.11	(0.98)
Age 64—WAIS Similarities (gi101re)	−0.05	(1.02)	0.00	(0.99)	−0.03	(1.01)	0.12	(0.95)
Age 72—WAIS Similarities (hi101re)	−0.03	(1.02)	−0.01	(0.97)	−0.03	(1.02)	0.10	(0.97)
Age 80—TICSm (q1i915re)	−0.03	(1.03)	0.01	(0.92)	−0.03	(1.01)	0.08	(1.04)
**Student- and School-Level Controls (Age 16)**								
ln of Family Income (piearl)	8.30	(0.76)	8.46	(0.71)	8.44	(0.73)	8.72	(0.71)
No. of Siblings (sibstt)	3.48	(2.47)	3.28	(2.44)	3.23	(2.43)	2.61	(2.10)
Father’s Education (bmfaedu)	9.46	(3.24)	9.65	(3.41)	9.95	(3.46)	10.28	(3.52)
Father’s Occupation (bmfoc1u)	30.46	(21.93)	34.14	(22.57)	34.42	(23.64)	40.59	(23.53)
Mother’s Education (bmmaedu)	10.38	(2.77)	10.32	(2.85)	10.69	(2.81)	10.56	(2.81)
School Mean [ln of Family Income]	8.30	(0.33)	8.46	(0.25)	8.44	(0.25)	8.72	(0.27)
School Mean of [Father’s Educ.]	9.49	(1.31)	9.68	(1.03)	9.99	(1.24)	10.31	(1.32)
School Mean of [Father’s Occ.]	30.47	(10.15)	34.20	(7.96)	34.55	(8.52)	40.70	(10.61)
School Mean of [Mother’s Educ.]	10.39	(1.03)	10.33	(0.84)	10.70	(0.92)	10.57	(1.01)
Size of Community (rlur57)	1.77	(0.81)	2.34	(0.80)	2.37	(0.98)	3.57	(0.83)

*Source*: Wisconsin Longitudinal Study. All cognitive outcome measures were standardized ~*N* (0.1). See *Materials and Methods* for more information about variables. Missing values for student-level control variables were imputed using chained equations; school-level control variables are aggregated up from student-level variables after imputation.

**Fig. 1.**
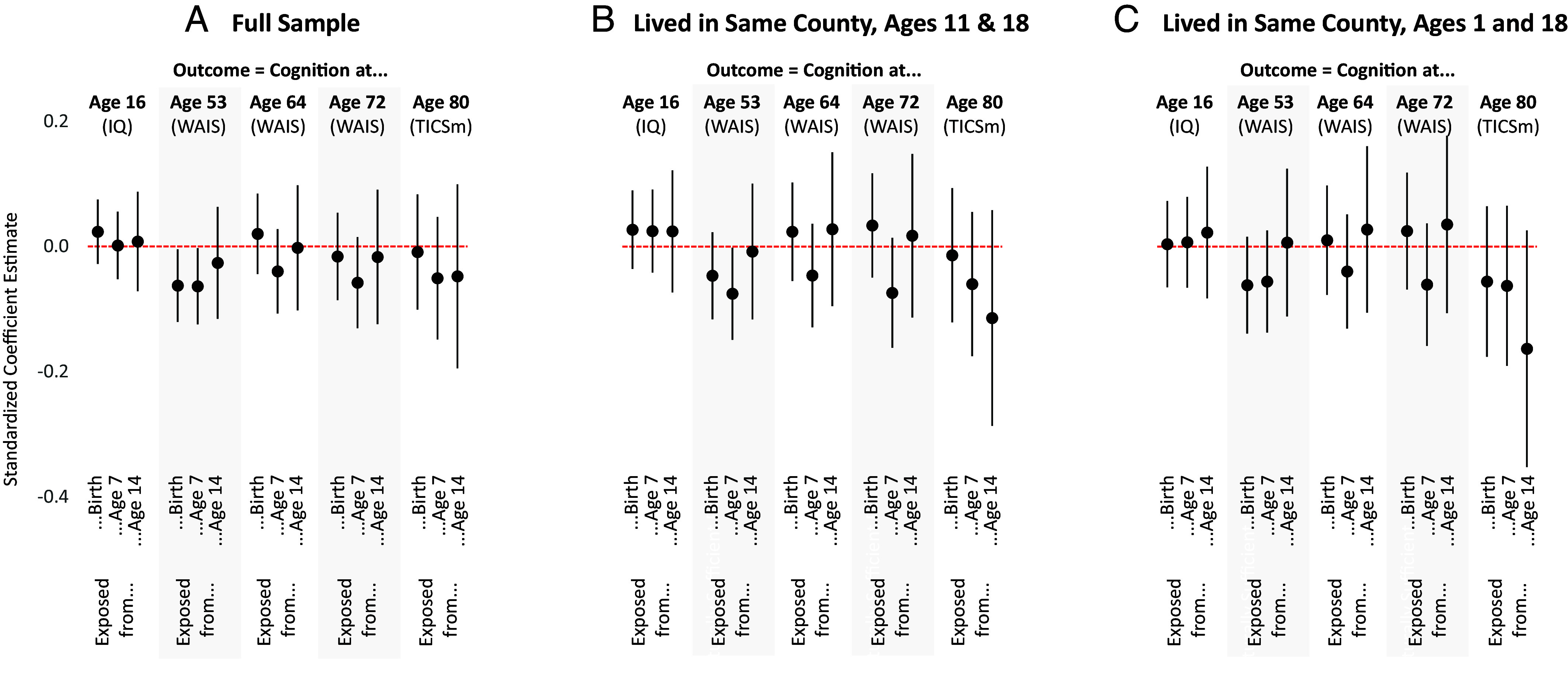
Panel *A* depicts results for the full WLS sample, while panels *B* and *C*, depict results for WLS participants who lived in the same county at ages 11 and 1, respectively.

Fig. 1 reports associations—after adjusting for student- and school-level confounders—between CWF and cognition across the life course. Panel (*A*) presents results for the full sample; Panels (*B*) and (*C*) restrict the sample to participants who lived in the same county as their high school at ages 11 and 1, respectively; this increases confidence that we are accurately characterizing which students were exposed to CWF for all or most of their lives.

As shown in Fig. 1, participants exposed to CWF did not perform significantly worse (or better) than their peers who were never exposed; this is true regardless of the age at which cognition was assessed and regardless of whether we restrict the sample to participants who did not move since ages 11 or 1. Only two of the 45 key coefficients are statistically distinguishable from zero—about what we would expect by chance.

## Discussion

We replicate and extend recent evidence (2) that CWF is not associated with reduced adolescent cognition. Our analysis improves on previous work, however, by using a measure of adolescent IQ (instead of academic achievement) and by more carefully considering adolescents’ duration of time in communities (and thus consistency of exposure to CWF). Like previous work, our research is limited by our inability to directly quantify adolescents’ consumption of fluoride (e.g., via urine samples).

We find no evidence that CWF is associated with lower adolescent IQ or cognition later in life.

## Materials and Methods

We utilize data from the WLS, which includes a 1/3 random sample (n = 10,317) of the Wisconsin high school graduating class of 1957. WLS data are available at https://www.ssc.wisc.edu/wlsresearch/data/, and were collected in accordance with protocols approved by the Institutional Review Board at the University of Wisconsin at Madison.

WLS data include the addresses of participants’ schools in 1957; because more than 90% of WLS records have been linked to the decennial US Censuses of 1940 and 1950—when sample members would have been ages 1 and 11, respectively—we are able to identify which sample members lived in their same communities over time.

Following the same methodology used by Warren et al. (2), we characterize sample members’ CWF exposure through age 14—prior to when IQ tests were typically administered—using information about CWF practices (6) and naturally occurring fluoride levels in untreated well water (7). Students were classified as exposed from birth if one or more untreated wells in their county had naturally sufficient fluoride levels. Otherwise, they were classified as being exposed to CWF beginning from the year in which their community began water fluoridation. See the *SI Appendix* available on the journal’s website for more details.

Adolescent IQ was measured using the standardized Henmon-Nelson test by schools in students’ freshman and junior years. Cognition at ages 53, 64, and 72 was measured using the similarities task of the Wechsler Adult Intelligence Scale (WAIS). Cognition at age 80 was measured using the Modified Telephone Interview for Cognitive Status (TICSm).

Control variables—introduced to account for selection into CWF—were collected in 1957 and include parental education, father’s occupation, family income, and size of community. We also include school means of family socioeconomic background measures. Unfortunately, the WLS did not collect information about dental care in adolescence or about non-water sources of fluoride exposure.

Missing values for student-level background measures were imputed using chained equations, with 20 imputed datasets. OLS regressions were estimated using “mi estimate” commands in Stata/SE 19.5.

## Supplementary Material

Appendix 01 (PDF)

## Data Availability

Some study data available WLS data are publicly and freely available at https://www.ssc.wisc.edu/wlsresearch/data/ (8). However, the replicate the current analyses, users will need to apply via the same website for restricted access in order to obtain detailed geographic information.
