# Supplementary material for: Municipal water fluoridation, adolescent IQ, and cognition across the life course: Evidence from the Wisconsin Longitudinal Study
Source: Proc Natl Acad Sci U S A. 2026 Apr 13;123(16):e2536005123. doi: 10.1073/pnas.2536005123 (PMC13099618; doi:10.1073/pnas.2536005123)
Supplement: Supplementary file 1 — Appendix 01 (PDF) [file pnas.2536005123.sapp.pdf]

Updated August 2025

PNAS

Supplementary Information for

Municipal Water Fluoridation, Adolescent IQ, and Cognition Across the Life Course: Evidence from the Wisconsin Longitudinal Study

Measure of Childhood Fluoride Exposure

WLS data include the geolocations of every Wisconsin secondary school in 1957 (when sample members were high school seniors, typically at about age 18). Records for about 90% of participants have been linked to the 1940 and 1950 decennial U.S. Censuses, providing information about participants' place of residence at the times of those enumerations (at about ages 1 and 11, respectively).

Using geolocations of WLS sample members' schools, we characterize sample members' fluoride exposure using archival data on (1) municipal water system fluoridation practices and (2) naturally occurring fluoride levels as measured in untreated well water.

First, we extracted records from the 1967 through 1993 editions of the U.S. Department of Health and Human Service's *Fluoridation Census* (1, 2). These records indicate which U.S. localities supplemented municipal drinking water with fluoride and the month and year in which they began doing so. Discrepancies between editions of the *Fluoridation Census* were reconciled via internet research on the history of cities' fluoridation efforts (using, for example, newspaper accounts and water utilities' web sites). Using information about each WLS panelist's date of birth, we are thus able to characterize the age at which sample members were first exposed to municipal water fluoridation.

Second, we utilized U.S. Geological Survey (USGS) data described by McMahon et al. (3) that characterize fluoride levels in untreated groundwater as measured in 38,105 wells in the United States between 1988 and 2017; data were extracted on March 25, 2025. Although these groundwater measures were mainly obtained in relatively recent years, the fact that fluoride levels in untreated water are determined by very slow-changing geologic processes means that these data also characterize fluoride levels that WLS panelists would have been exposed to early in life.

In these data, each well is assigned a USGS identifier (ID). The USGS ID consists of 15 digits: The first six digits denote the degrees, minutes, and seconds of latitude; the next seven digits denote degrees, minutes, and seconds of longitude; and the last two digits are a sequential number for wells within a 1-second grid. If the latitude-longitude coordinates for a well are the same, a sequential number (e.g., "01" or "02") is assigned. We converted the latitude and longitude of each well into decimals using the formula: Decimal Latitude/Longitude = degrees + (minutes/60) + (seconds/3600). Next, we used the add-on module opencagegeo in Stata 18 to convert latitudes and longitudes into exact street addresses and to determine city, county, and state of each well's location.

We linked each school's location to fluoride levels from the well closest to the school. Students were classified as exposed from birth if one or more untreated wells in their county had naturally sufficient fluoride levels. Otherwise, they were classified as being exposed to CWF beginning from the year in which their community began water fluoridation (if ever).

The U.S. Centers for Disease Control and Prevention (4) currently recommends community water fluoridation levels of 0.7 mg/L. Using all the information described above, we construct a single measure of fluoride exposure that can take on four possible values. First, WLS participants were classified as

consistently exposed to insufficient levels of fluoride if they lived in places that (a) did not utilize municipal water fluoridation and (b) lived in counties with no well that had naturally occurring fluoride levels of 0.7 mg/L or higher. Second, WLS participants were classified as *consistently exposed to sufficient levels of fluoride* if they lived in counties that had at least one well with naturally occurring fluoride levels at or above 0.7 mg/L. Third, students were classified as *exposed to sufficient levels of fluoride after age 7* if they lived in places that implemented municipal water fluoridation after they turned 7 but before they turned 14; here, we assume that places that implemented municipal water fluoridation ─ all in or after 1946 ─ had naturally occurring fluoride levels below 0.7 mg/L prior to fluoridation. Fourth, students were classified as *exposed to sufficient levels of fluoride after age 14* if they lived in places that implemented municipal water fluoridation after they turned 14; we include this category as a robustness check: If we had found that implementing CWF after participants took IQ tests was associated with scores on that test, we would have been concerned about residual confounding.

We perform three sets of empirical analyses. FIRST, we begin by assuming that (1) students lived geographically close to their secondary schools at age ~18 and (2) students lived in the same community for the entirety of their childhoods through 1957 (or about age 18). The former assumption is reasonable given that WLS students attended secondary school in an era before the widespread implementation of school choice, magnet school, and other enrollment policies that have subsequently reduced the percentage of students who attend neighborhood schools. We relax the latter assumption by considering students' residential locations at about age 1 and about age 11 in supplementary analyses. So, in our SECOND and THIRD sets of analyses we restrict the sample to WLS sample members who lived in the same county at ages 11 and 18 and to WLS sample members who lived in the same county at ages 1 and 18, respectively. Although the second and third sets of analyses have smaller sample sizes (and lack some external validity), we are more confident in those analyses that we are accurately characterizing exposure to fluoride through municipal and well water.

References

1. U.S. Department of Health and Human Services, *Fluoridation Census* (U.S. Centers for Disease Control and Prevention, 1993).

2. U.S. Department of Health, Education, and Welfare, *Fluoridation Census, 1966* (U.S. Department of Health, Education, and Welfare, 1967).

3. P. B. McMahon, C. J. Brown, T. D. Johnson, K. Belitz, B. D. Lindsey, Fluoride occurrence in United States groundwater. *Science of the Total Environment* **732**, 139217 (2020).

4. T. J. Boehmer, Community water fluoridation levels to promote effectiveness and safety in oral health—United States, 2016–2021. *MMWR. Morbidity and Mortality Weekly Report* **72** (2023).
